# Fatigue, depression, and impaired health-related quality of life in patients with vascular liver diseases: A multicentric European study^☆^

**DOI:** 10.1016/j.jhepr.2026.101861

**Published:** 2026-04-17

**Authors:** Clémence Ramier, Virginia Hernandez-Gea, Laure Elkrief, Annalisa Berzigotti, Andrea De Gottardi, Antonina Antonenko, Audrey Payancé, Pierre-Emmanuel Rautou, Terhi Kangas, Hadewijch Vandenheede, Katrien Vanthomme, Gaël Brulé, Agnes Dumas, Aurélie Plessier, VALDIG consortium

**Affiliations:** 1 INSERM, Aix Marseille University, IRD, ISSPAM, SESSTIM, Sciences Economiques & Sociales de la Santé & Traitement de l’Information Médicale, Equipe CALIPSO, Marseille, France; 2 Barcelona Hepatic Hemodynamic Laboratory, Liver Unit, Fundació de Recerca Clínic Barcelona (FRCB-IDIABPS), CIBEREHD (Centro de Investigación Biomédica en Red Enfermedades Hepáticas y Digestivas), Health Care Provider of the European Reference Network on Rare Liver Disorders (ERN-RareLiver), University of Barcelona, Barcelona, Spain; 3 Departament de Medicina i Ciències de la Salut, Universitat de Barcelona, 08036 Barcelona, Spain; 4 Université Paris-Cité, Inserm, Centre de Recherche sur l'Inflammation, UMR 1149, Paris, France; 5 Service D’Hépato-Gastroentérologie, Centre de Référence des Maladies Vasculaires du Foie, FILFOIE, ERN RARE-LIVER, CHRU de Tours, Tours, France; 6 Department of Visceral Surgery and Medicine, Inselspital, Bern University Hospital, University of Bern, Bern, Switzerland; 7 Gastroenterology and Hepatology, Luzerner Kantonsspital, Lucerne, Switzerland; 8 Gastroenterology and Hepatology, Ente Ospedaliero Cantonale, Lugano, Switzerland; 9 AP-HP, Hôpital Beaujon, Service d'Hépatologie, DMU DIGEST, Centre de Référence des Maladies Vasculaires du Foie, FILFOIE, ERN RARE-LIVER, Clichy, France; 10 Vrije Universiteit Brussel, Brussels Institute for Social and Population Studies (BRISPO), Brussel, Belgium; 11 Ghent University, Department of Public Health and Primary Care, Ghent, Belgium; 12 Geneva School of Health Sciences, University of Applied Sciences and Arts of Western Switzerland, Geneva, Switzerland

**Keywords:** Quality of life, Rare disease, Vascular diseases, Liver disease, Patient-reported outcomes, Fatigue, Depression, Portal vein, Budd-Chiari syndrome, Cost of illness

## Abstract

**Background & Aims:**

Patient-reported outcomes (PROs) are scarce in vascular liver diseases (VLD), including portal vein thrombosis (PVT) and Budd-Chiari syndrome (BCS). This study aimed to assess health-related quality of life (HRQoL), fatigue, and depressive symptoms in patients with VLD and to identify associated factors.

**Methods:**

We performed a multicentre cross-sectional study in France, Spain, and Switzerland. Patients with PVT or BCS were asked to fill out validated questionnaires assessing health-related quality of life (HRQoL; EQ-5D-5L), fatigue (Modified Fatigue Impact Scale - short form; MFIS-5), and depressive symptoms (Patient Health Questionnaire-8; PHQ-8). Clinical and sociodemographic data were collected. Linear and logistic regression analyses were used to identify factors associated with the three PROs. Comparisons with the general population were conducted.

**Results:**

Among 1,136 eligible patients, 488 respondents completed the three PROs. The mean ± SD HRQoL score was 0.885 ± 0.146, significantly lower than that of the French general population but comparable to the Spanish general population. Depressive symptoms were reported in 24.8% of patients (*vs.* 9.8% and 3.7% in the French and Spanish general populations, respectively). After indirect standardisation for gender, age, and education, the prevalence of depressive symptoms was three times higher in French patients than in the French general population. Lower HRQoL and greater fatigue were significantly associated with female gender, financial difficulties, self-reported comorbidities, and a history of hepatic encephalopathy on multivariable analysis. Depressive symptoms were associated with female gender, financial difficulties, and antiphospholipid syndrome, whereas anticoagulation therapy was associated with better outcomes.

**Conclusions:**

The disease burden in patients with VLD is significant, with impaired HRQoL and high rates of fatigue and depressive symptoms. Hepatic encephalopathy, gender, and socioeconomic factors are key drivers**,** emphasising the need for comprehensive care approaches that integrate psychological and social support with clinical management.

**Impact and implications:**

This study provides new insight into the disease burden of VLDs by quantifying the prevalence of impaired HRQoL, fatigue, and depressive symptoms in patients with PVT and BCS. Our findings show that female gender and socioeconomic factors, especially financial difficulties, have a more important role compared with clinical severity in predicting PRO measures. These results show the association between anticoagulation therapy and lower levels of fatigue and depression, and the need to expand current care models beyond medical treatment to include psychological support and patient education programs to manage fatigue. Future longitudinal studies are needed to monitor the long-term evolution of these outcomes.

## Highlights

• HRQoL impairment was mainly linked to socioeconomic and not clinical factors.
• Women and patients with financial difficulties reported poorer HRQoL and more fatigue.
• Oral anticoagulation was associated with lower levels of fatigue and depression.
• Depression was three times higher in patients with VLD than in the general population.
• Integrating psychological support and fatigue care in VLD management is needed.

## Graphical abstract

**Figure:**
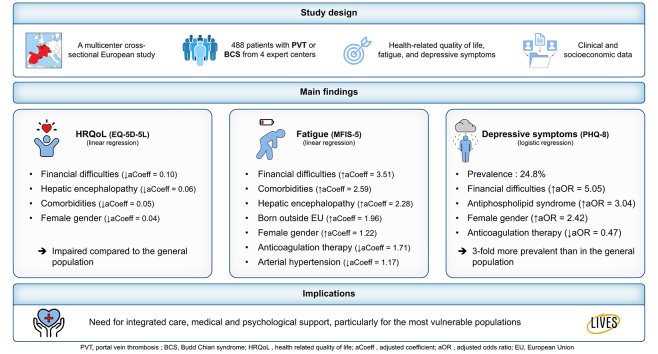

## Introduction

Budd-Chiari syndrome (BCS) and portal vein thrombosis (PVT) are rare vascular liver diseases (VLD) that mainly affect young and economically active individuals.^1^^,^^2^ Their estimated prevalence in Europe is 1.4–4 per million for BCS and from two to four per 100,000 for PVT.^1^^,^^3^ These conditions are frequently associated with both inherited and acquired prothrombotic risk factors^2^^,^^4^ requiring multidisciplinary care. Women of reproductive age represent up to 50% of BCS and 20% of PVT cases, often experiencing fertility and pregnancy-related complications.^5^ Management usually requires long-term anticoagulation and, in many cases, invasive interventions, such as transjugular intrahepatic porto-systemic shunts (TIPS) or surgery.^2^ Complications during follow-up include portal hypertension (PH), PH-related issues, and recurrent thrombosis. Moreover, many patients present underlying prothrombotic disorders that can also progress during follow-up.^6^^,^^7^ Although survival has improved significantly in recent years, with 5-year survival rates approaching 80% in BCS and 85% in PVT,^8^^,^^9^ the long-term disease burden remains significant, with increased hospital admissions over the past decade.^10^ Once diagnosed and stabilised, the disease often becomes invisible to the patient’s social and professional environment, potentially masking ongoing physical and psychological challenges.

Patient-reported outcomes (PROs) can be used to assess many health-related issues experienced by patients. One such issue is health-related quality of life (HRQoL), which is usually defined as the impact of health on daily functioning.^11^ HRQoL has become a crucial outcome measure in chronic liver diseases, where symptoms, such as fatigue and depression, are common and can significantly impair daily functioning.^12^^,^^13^ Although HRQoL has been extensively studied in patients with cirrhosis,^14^^,^^15^ patients with BCS or non-cirrhotic PVT (NC-PVT) differ markedly in terms of age, long-term anticoagulation, associated comorbidities, and fertility-related issues. Data for VLD are limited, with only one published study based on administrative data^16^ and no studies incorporating PROs.

Thus, the goal of this multicentre European study was to assess HRQoL, fatigue, and depression in patients with BCS or NC-PVT, and to identify associated factors.

## Methods

### Population and data collection

This cross-sectional study included adults (aged ≥18 years) who received treatment for NC-PVT or BCS at four specialised centres of the Vascular Liver Disease Group (VALDIG) located in France, Spain, and Switzerland. Detailed clinical, laboratory, and imaging data from patients with VLDs were prospectively collected in a regularly updated registry at all centres for accurate follow-up of all enrolled patients. The most recent contact information was collected from the clinical centre for all patients included in the registry. Patients with cirrhotic PVT were excluded from the study because the natural history and management of this condition are different from those for non-cirrhotic PVT.

Clinical data were extracted from this registry. In 2024 a self-administered online questionnaire collecting sociodemographic, socioeconomic, and PROs was proposed to all eligible patients (≥18 years old and living at the time of the study) in French, Spanish, and German, with up to two reminders sent in case of a nonresponse. Validated translations of the three HRQoL scales were used (EQ-5D, Patient Health Questionnaire-9 [PHQ-9], and Modified Fatigue Impact Scale - short form [MFIS-5]). The sociodemographic and socioeconomic items of the questionnaire were the same as those used in general European population surveys. All other translations were reviewed and discussed by native speakers (members of patient organizations and members of the clinical research team).

The LIVES quantitative study was approved by the ethics committee of each site in France (Inserm’s ethics committee, CEEI-IRB [IRB00003888], approval 22-940,), Spain (Hospital Clínic de Barcelona, Comité de Ética de la Investigación con medicamentos, approval HCB/2022/0064), and Switzerland (Cantonal Ethics Committee of Bern, approval 2023-00556).

### Study outcomes

We used three distinct outcomes related to quality of life, using validated scales to assess overall HRQoL and specific symptoms, in particular fatigue and depressive symptoms. These scales were chosen according to the PRO guidelines from the European Reference Network on Rare Liver Disease to enhance the comparability of data. Furthermore, we examined fatigue levels and depressive symptoms because these outcomes are known to be prevalent in individuals with rare diseases and frequently reported by patients with VLD, as seen in medical observations.

HRQoL was evaluated using the EQ-5D-5L questionnaire,^17^ a validated standardised instrument that assesses five dimensions of health: mobility, self-care, usual activities, pain/discomfort, and anxiety/depression. A single summary index was created by applying dimension- and country-specific weights to the patient responses, using validated index values for France^18^ and Spain,^19^ and supra-national European values for Switzerland.^20^ Higher index scores reflect better HRQoL.

Fatigue was measured using MFIS-5,^21^ a condensed version of the original 40-item Fatigue Impact Scale,^22^ which has been validated in several liver diseases.^23^ This five-item questionnaire assesses the impact of fatigue on physical, cognitive, and psychosocial functioning. Total scores range from 0 to 20, with higher scores indicating greater fatigue-related impairment.

Depressive symptoms were assessed using the PHQ-9,^24^ a nine-item self-administered instrument evaluating symptom frequency over the previous 2 weeks. An eight-item version with similar psychometric properties and normative scores is available from European general population surveys; thus, we restricted the depressive symptoms evaluation to the PHQ-8 scale instead of the PHQ-9 scale. Each item is rated on a 4-point Likert scale, yielding a total score of 0–24. A score of ≥10 was used to define depressive symptoms.^24^

The three validated scales were provided in Table S1.

### Explanatory variables

Clinical variables included diagnosis-specific data, VLD-related complications, associated diseases, BCS prognosis scores, and therapeutic strategies. Diagnosis-specific data included the type of VLD (NC-PVT or BCS), age at diagnosis, and time since diagnosis. VLD-related complications included impaired fertility, abdominal pain, ascites, oesophageal or gastric varices, gastrointestinal bleeding, history of overt hepatic encephalopathy (HE; i.e. West grade 2 onward), thrombotic events, and liver cancer. Impaired fertility was only self-reported. The following question was asked: ‘We know that vascular liver diseases can induce fertility impairment. Have you personally ever had difficulty having a child (miscarriages, use of assisted reproductive techniques)?’. Associated diseases covered both inherited and acquired prothrombotic disorders (e.g. myeloproliferative leukaemia [MPL], antiphospholipid syndrome [APS], paroxysmal nocturnal haemoglobinuria [PNH], and Behcet’s disease), as well as other conditions, including self-reported comorbidities (Fig. S1), diabetes, arterial hypertension, and anaemia (haemoglobin <10 g/dl). Prognostic scores for BCS included the Child-Pugh score, the Rotterdam score, and Clichy’s criteria. Therapeutic strategies included medical therapies (e.g. anticoagulants, diuretics, antiplatelet agents, and beta-blockers), as well as follow-up in an anticoagulant clinic, interventional radiology, or surgical procedures (e.g. angioplasty, stenting, TIPS, or shunt surgery), and liver transplantation.

All clinical variables were obtained from the VALDIG registry between the time of inclusion and completion of the questionnaire, to reflect a history of clinical events between these two time points. The only exception was BCS prognostic scores, which were based on the latest available data before completing the questionnaire, and for impaired fertility and self-reported comorbidities, which were obtained from the questionnaire.

Sociodemographic and socioeconomic variables included gender, age at questionnaire, country of birth, living with a partner, educational level, and perceived financial difficulties. Country of birth was dichotomised into European countries and non-European countries. Educational level was classified using the International Standard Classification of Education (ISCED) into three categories: low (ISCED 0–2), moderate (ISCED 3–4), and high (ISCED 5–8). Financial difficulties were assessed with the question: ‘Thinking about your household's monthly income, would you say that your household manages to make ends meet?’. Responses were grouped into three categories: (i) no difficulty (‘very easy’ or ‘easy’), (ii) few difficulties (‘relatively easy’ or ‘with some difficulty’), and (iii) a lot of difficulties (‘with difficulty’ or ‘with great difficulty’).

### Statistical analyses

Respondents and nonrespondents were compared using the chi-square or Fisher’s exact tests for categorical variables, and the Kruskal-Wallis test for continuous variables. We arbitrarily defined two cut-offs for the description of the MFIS-5 scale: ≥15/20, corresponding to the highest quarter of the score; and ≥18/20, reflecting a value close to the maximum. We used the continuous score for analysis.

Multivariable regressions were performed to identify factors associated with each outcome (linear for HRQoL and fatigue, which are continuous outcomes, and logistic for depressive symptoms, which is a binary outcome). Explanatory variables with a univariable *p* <0.20 were considered eligible for multivariable modelling. Final models were selected using a backward stepwise approach with significance set at 5%. Robust sandwich estimators were used to calculate variance and CIs.

Supplementary analyses were performed in the EQ-5D-5L and MFIS-5 subdomains to further explore the specific impact of significant predictors. Explanatory analyses were also performed in all female patients as well as in female patients of childbearing age (20–45 years) to assess the association between impaired fertility and each outcome.

HRQoL scores were compared with normative values from the general populations in France^25^ and Spain,^26^ both overall and by gender, using Student’s *t* test. The prevalence of depressive symptoms was compared with that of the general population using data from the 2019 European Health Interview Survey.^27^ Given sample size limitations, this comparison was restricted to French participants aged 25–74 years with moderate or high educational levels. The comparison was performed using indirect standardisation by gender, age, and educational level, and a standardised incidence ratio (SIR) of depressive symptoms was calculated for this subgroup.

All analyses were performed with Stata version 19.0 for Windows software (StataCorp LP, College Station, TX, USA).

## Results

**Fig. 1:**
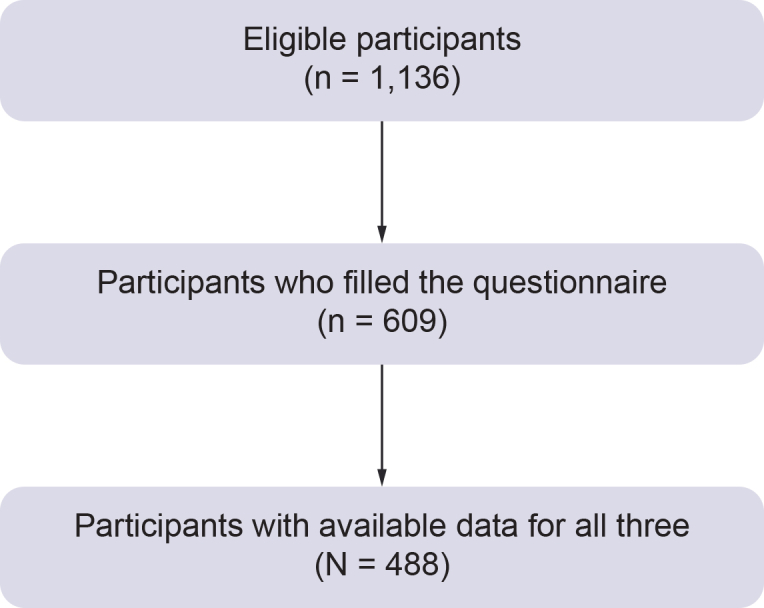
Flowchart of the study population selection. PRO, patient-reported outcome.

Patients with missing data on the EQ-5D-5L, MFIS-5, or PHQ-8 scales were excluded. The flowchart of the study population selection is illustrated in Fig. 1.

### Comparison between respondents and nonrespondents

In total, 609/1,136 (53.6%) patients completed at least part of the questionnaire, and 488 (43.0%) completed all three PRO scales. Characteristics of respondents *vs.* nonrespondents are shown in Table S2. Respondents had more BCS (*p* = 0.031), fewer oesophageal varices (*p* = 0.011), and more thrombotic events (*p* = 0.001). They more often had associated diseases, particularly MPL (*p* = 0.044) and PNH (*p* = 0.030). Liver function was poorer in respondents, with a higher proportion of Rotterdam score class II and III (*p* = 0.022) and a higher proportion of Child-Pugh score class B (*p* = 0.036), suggesting more complex cases. Respondents were also more frequently treated with anticoagulation therapy (*p* = 0.008).

### Study population characteristics

The study population included 488 patients, 53.1% men, median age 53 years (IQR: 42.5–63.5) (Table 1). Most patients had NC-PVT (75.0%; 75.7% in France, 68.6% in Spain, and 84.2% in Switzerland) and a high level of education (55.5%). In addition, 264 patients (58.9%) reported having comorbidities in the self-administered questionnaire, most frequently, associated with VLD (26.7%), related to the metabolic syndrome (9.6%), psychiatric disorders (6.7%), or cancer (5.8%). VLD-related complications were frequent: abdominal pain (331; 71.5%), ascites (345; 74.5%), and oesophageal varices (242; 53.4%), although a history of overt HE was rare (23; 5.4%). Nearly 80% (390 patients) received anticoagulation therapy (Table 1).

**Table 1:** Study population characteristics.

Explanatory variables **(% of missing values)**	Study population (N = 488)
	N (%) or median (IQR)
**Sociodemographic and economic characteristics**	
**Gender** (0)	
Men	259 (53.1)
Women	229 (46.9)
**Age** (0)	53 [42.5–63.5]
**Country of birth** (1.6)	
EU	416 (86.7)
Non-EU	64 (13.3)
**Living with a partner** (3.9)	322 (68.7)
**Educational level** (0.6)	
Low	27 (5.6)
Moderate	189 (39.0)
High	269 (55.5)
**Financial difficulties** (0.4)	
No	178 (36.6)
Few	241 (49.6)
A lot	67 (13.8)

**Diagnosis-specific characteristics**	
**Diagnosis** (0)	
PVT	366 (75.0)
BCS	122 (25.0)
**Age at diagnosis** (0)	43 (31–54)
**Time since diagnosis***(in years)* (0)	8.7 [4.6–14.3]
**Time since diagnosis** (0)	8.7 [4.6–14.3]
≥2 years	453 (92.8)
<2 years	35 (7.2)
**Time since last clinical examination** (in years) (0)	0.8 [0.4–1.4]

**History of VLD-related complications**	
**Impaired fertility** (8.2)	64 (14.3)
**Abdominal pain** (5.1)	331 (71.5)
**Ascites** (5.1)	345 (74.5)
**Oesophageal varices** (7.2)	242 (53.4)
**Gastric varices** (7.4)	55 (12.2)
**Gastrointestinal bleeding** (5.3)	76 (16.5)
**History of overt hepatic encephalopathy** (12.3)	23 (5.4)
**Thrombotic event** (0.8)	206 (42.6)
**Liver cancer** (12.3)	7 (1.6)

**History of associated diseases**	
**Self-reported comorbidities** (8.2)	264 (58.9)
**Diabetes** (5.3)	34 (7.4)
**Arterial hypertension** (5.7)	75 (16.3)
**Anaemia** (7.8)	47 (10.4)
**Myeloproliferative leukaemia** (6.4)	127 (27.8)
**Antiphospholipid syndrome** (9.2)	19 (4.3)
**Paroxysmal nocturnal haemoglobinuria** (17.2)	12 (3.0)
**Behcet’s disease** (8.2)	18 (4.0)
**Factor V Leiden mutation** (10.0)	39 (8.9)
**Prothrombin G20210A mutation** (10.7)	38 (8.7)

**Last available prognosis scores for BCS**	
**Child-Pugh score** (3.1)	
PVT	366 (77.4)
Class A	29 (6.1)
Class B	69 (14.6)
Class C	9 (1.9)
**Rotterdam score** (4.3)	
PVT	366 (78.4)
Class I	34 (7.3)
Class II	31 (6.6)
Class III	36 (7.7)
**Clichy score** (3.3)	
PVT	366 (77.5)
Class I	82 (17.4)
Class II	24 (5.1)

**History of therapeutic strategies**	
**Interventional radiology or surgical procedures**∗ (12.3)	79 (18.5)
**Liver transplant** (12.3)	6 (1.4)
**Anticoagulation therapy** (0)	390 (79.9)
**Diuretic therapy** (0)	55 (11.3)
**Antiplatelet therapy** (0)	25 (5.1)
**Beta-blocker therapy** (0)	103 (21.1)
**Follow-up in anticoagulant clinic** (0)	123 (25.2)

BCS, Budd-Chiari syndrome; EU, European Union; PVT, portal vein thrombosis; TIPS, transjugular intrahepatic porto-systemic shunt.

∗ Interventional radiology or surgical procedures included angioplasty, stenting, TIPS, or shunt surgery.

The mean HRQoL score was 0.885 ± 0.146 (mean ± SD). Only 18.5% of participants reported a health status of ‘11111’ (full health), comprising 18.1% of French participants, 22.9% of Spanish participants, and 15.8% of Swiss participant. The HRQoL of French participants was lower than that of the French general population (0.885 ± 0.155 *vs.* 0.905 ± 0.158, *p* = 0.013), especially in women (0.856 ± 0.185 *vs.* 0.895 ± 0.166, *p* = 0.001). No significant difference was observed in the Spanish population (0.886 ± 0.089 *vs.* 0.897 ± 0.212, *p* = 0.664).

The mean fatigue score was 8.61 ± 5.25 (mean ± SD), with a median (IQR) score of 9 (5–12). Nearly 15% of patients reported a score of at least 15/20, and 24 (4.9%) patients reported a score of at least 18/20 (nonvalidated clinical thresholds). The median fatigue scores of patients with depressive symptoms were nearly twice as high as patients without (13 [10–16] *vs.* 7 [3–11], *p* = 0.001).

The prevalence of depressive symptoms was 24.8% (25.1%, 25.7%, and 15.8% in French, Spanish, and Swiss participants, respectively, *p* = 0.738), and 15% of patients reported recent suicidal ideation. Reported depressive symptoms in French participants aged 25–74 years with moderate and high educational levels were 27.6% and 25.2%, respectively. After indirect standardisation, patients with moderate education and high education had a 3.2-fold (SIR 95% CI: 3.21 [2.32–4.34]) and 3.8-fold (SIR: 3.79 [2.84–4.95]) higher prevalence, respectively, of depressive symptoms compared with the general population.

### Factors associated with PROs

Univariable analyses are presented in Table S3 for all three PRO scales.

**Fig. 2:**
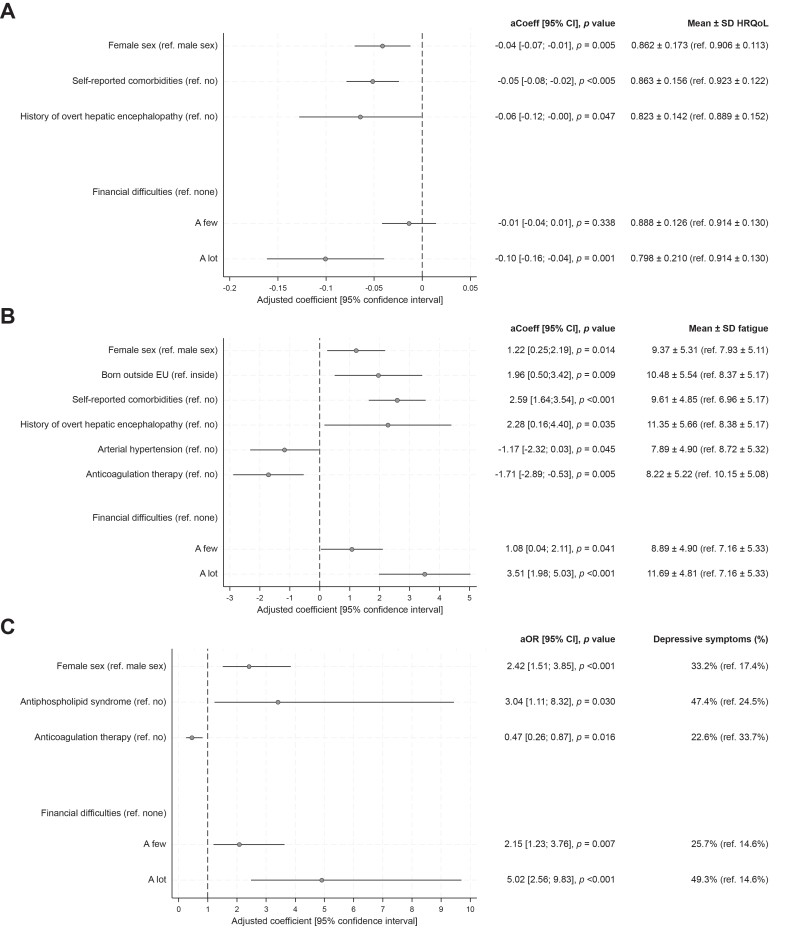
Factors associated with HRQoL (EQ-5D-5L), fatigue (MFIS-5) and depressive symptoms (PHQ-8) in multivariable analyses (linear and logistic regressions). (A) Factors associated with HRQoL (EQ-5D-5L) – multivariable linear regression (n = 391, 97 patients omitted because of missing data for adjusted variables). (B) Factors associated with fatigue (MFIS-5) – multivariable linear regression (n = 386, 102 patients omitted because of missing data for adjusted variables). (C) Factors associated with depressive symptoms (PHQ-8) – multivariable logistic regression (n = 441, 47 patients omitted because of missing data for adjusted variables). aCoeff, adjusted coefficient; aOR, adjusted odds ratio; EU, European Union; HRQoL, health-related quality of life; MFIS-5, Modified Fatigue Impact Scale - short form; PHQ-9, Patient Health Questionnaire-9. Level of significance: *p* <0.05.

Multivariable analysis of the HRQoL (Fig. 2A) showed that female gender (*p* = 0.005), a lot of financial difficulties (*p* = 0.001), self-reported comorbidities (*p* <0.001) and a history of overt HE (*p* = 0.047) were associated with a lower HRQoL, with corresponding decreases of 0.04, 0.10, 0.05, and 0.06, respectively. Sensitivity analyses showed that all these factors, except a history of overt HE, influenced four EQ-5D-5L subdomains (mobility, usual activities, pain/discomfort, and anxiety/depression), whereas HE only affected anxiety/depression (Table S4).

Fatigue was significantly associated with female gender (*p* = 0.014), being born outside the EU (*p* = 0.009), and financial difficulties (*p* = 0.041 for few; *p* <0.001 for a lot of difficulties) (Fig. 2B), corresponding to an increase of fatigue levels of 1.22, 1.96, 1.08, and 3.51, respectively. Self-reported comorbidities (*p* <0.001) and a history of overt HE (*p* = 0.035) significantly increased fatigue levels by 2.59 and 2.28, respectively. By contrast, arterial hypertension (*p* = 0.045) and anticoagulation (*p* = 0.005) were inversely associated with fatigue and decreased fatigue levels by 1.17 and 1.71, respectively. The results for female gender, financial difficulties, self-reported comorbidities, and anticoagulation therapy were consistent across all three of the MFIS-5 domains (Table S5). Being born outside the EU was associated with both the physical and psychosocial domains. A history of overt HE and arterial hypertension were only associated with the cognitive domain.

In terms of depressive symptoms, multivariable logistic regression identified female gender and financial difficulties as strong predictors of depressive symptoms. The odds of depressive symptoms was twice as high in women as in men (adjusted odds ratio [aOR] 2.42; 95% CI: 1.51–3.85, *p* <0.001). The odds of depressive symptoms was twice as high in patients with few financial difficulties than in those without (aOR 2.15; 95% CI: 1.23–3.76, *p* = 0.007) and was five times higher in those with significant financial difficulties (aOR 5.02; 95% CI: 2.56–9.83, *p* <0.001) (Fig. 2C). The odds of depressive symptoms was three times higher in patients with APS (aOR 3.04; 95% CI: 1.11-8.32, *p* = 0.030), whereas the odds in those receiving anticoagulation therapy was decreased by 53% (aOR 0.47; 95% CI: 0.26-0.87, *p* = 0.016).

After adjustment for age and self-reported comorbidities, impaired fertility in women of childbearing age (n = 97, data not shown) was associated with a lower HRQoL (aCoeff: −0.16; 95% CI: −0.29 to −0.03], *p* = 0.020), greater fatigue (aCoeff: 2.42; 95% CI: 0.01–4.84, *p* = 0.049) and a greater odds of depressive symptoms (aOR: 3.47; 95% CI: 1.13–10.69, *p* = 0.030). In the overall female population, only depressive symptoms remained significantly associated with impaired fertility (aOR: 2.02; 95% CI: 1.08–4.00, *p* = 0.044) (data not shown).

## Discussion

The disease burden of VLD has remained largely unexplored. This multicentre European study is the first to fill the knowledge gap on the impact of VLD on PROs among patients with NC-PVT or BCS. Our findings show that HRQoL was significantly poorer in patients with VLD, mainly associated with female gender and socioeconomic factors and, to a lesser extent with clinical factors such as self-reported comorbidities and a history of overt HE.

One important finding in our study was the prevalence of depressive symptoms, which was significantly higher than that of the general population. Chronic liver disease appears to be a risk factor for depression, affecting 15–30% of patients,^28^^,^^29^ as well as those with rare chronic diseases, with an estimated global prevalence of depressive symptoms of 13.1%.^30^ This high prevalence of depression might the result of the influence of the delay in diagnosis on psychological outcomes.^31^ In support of our findings, a large German study found PVT to be independently associated with a two-fold increase in depression and anxiety.^16^

Fatigue is closely linked to depressive symptoms and impaired HRQoL. As previously shown, fatigue was a strong driver of HRQoL impairment.^32^ Chronic fatigue affects 50–85% of patients with liver disease and often improves with treatment of the underlying cause.^33^ However, little is known about its epidemiology, pathogenesis, or treatment in liver diseases, and nothing is known for VLD. Fatigue pathogenesis in cirrhosis is multifactorial and is not necessarily correlated with the severity of liver disease.^34^ In our cohort, fatigue was associated with a history of overt HE and self-reported comorbidities. This is similar to findings in studies of pretransplant cirrhosis, where HE was associated with both physical and psychosocial fatigue subdomains.^35^ Our data suggest that overt HE more specifically affects cognitive fatigue. A history of overt HE was also found to be a key clinical factor of HRQoL, particularly in the anxiety/depression subdomain. This is consistent with previous reports in patients with cirrhosis, where HE was associated with poorer mental, but not physical HRQoL.^36^ Moreover, evidence suggests that overt HE can result in impaired health function, including cognitive function, even after it has been resolved and the patient’s clinical status appears to be satisfactory.^36^ Furthermore, circadian rhythm disturbances and poor sleep quality are common in patients with cirrhosis because of altered melatonin production, temperature regulation, and exposure to light.^37^ Thus, the impact of circadian rhythm disturbances should be assessed in this setting.

Social determinants also had a role. Similar to other studies associating female gender and low income with poorer HRQoL in patients with rare diseases^38^ or chronic diseases,^39^ financial difficulties and female gender were associated with greater fatigue in our study. Fatigue in the general population is known to be 40% more prevalent in women than in men,^40^ and women consistently report poorer mental health compared with men, possibly because of inequalities in the division of unpaid domestic work.^41^ However, our findings also show the significant impact of fertility difficulties on the mental health and overall well-being of women with a VLD. Financial difficulties were also consistently associated with all PROs, probably reflecting the social inequalities in health that are observed in the general population^42^^,^^43^ or in patients with liver diseases.^44^ Psychological distress from financial difficulties, with low income as a major risk factor, is prevalent among patients with chronic liver disease (36–63%) and can lead to anxiety and depression.^45^ Even if we ignore the cause of these financial difficulties, our findings emphasise the importance of proportionate universalism, which advocates scaled interventions to target deprived patients, thus reducing economic disparities in HRQoL in patients with VLDs.

Anticoagulation was associated with lower levels of fatigue and depression. Similar results were found in patients treated with anticoagulation for atrial fibrillation, with a significant overall benefit with oral anticoagulation.^46^ Patient empowerment for the management of oral anticoagulation therapy was associated with decreased distress and enhanced HRQoL.^47^^,^^48^ Although we did not observe a significant difference in outcomes in the patients followed in an anticoagulation clinic, the impact of patient education on HRQoL warrants further exploration. Furthermore, national and EU-level guidelines encouraging access to specialised anticoagulation services could improve both mental and physical outcomes. Although anticoagulation appeared to effectively reduce worry about thrombosis and improve empowerment, there is also a risk of confounding because of an indication and survival bias. Indeed, patients receiving long-term anticoagulation therapy might have been those who survived and remained adherent during follow-up, leading to a possible improvement in liver disease. This could also be true for the protective impact of arterial hypertension on fatigue because patients with arterial hypertension are regularly followed up and might have better overall cardiovascular control.

Self-reported comorbidities were strongly associated with impaired HRQoL in all EQ-5D-5L subdomains. Similar results were described for deep venous thrombosis, with impaired HRQoL in the presence of comorbidities.^49^ A recent systematic review of the literature estimated that the minimal important difference for the EQ-5D-5L mean score ranged from 0.005 to 0.410, with a median of 0.065 for various conditions but not for liver diseases.^50^ In line with this review, HRQoL impairment caused by a history of overt HE (0.06 decrease) and extreme financial difficulties (0.10 decrease) were close to or higher than the estimated median minimal important difference, highlighting their clinical importance. By contrast, despite the significant association of female gender and self-reported comorbidities with impaired HRQoL, these factors appeared less clinically relevant.

Despite the significant burden of HRQoL reported in our cohort, mean HRQoL scores were higher than those observed in a European cohort of patients with autoimmune liver diseases, (0.885 *vs.* 0.75).^51^ Nevertheless, women consistently reported lower HRQoL compared with men in both studies as well as in the general population.^25^^,^^26^ As our subgroup analysis showed, fertility issues might have contributed to this gender disparity and deserve further investigation. In particular, women with VLD are known to experience a high prevalence of miscarriage (20% for PVT and 29% for BCS^5^ *vs.* 15.3% in the general population^52^), which could affect their PROs.

The burden of fatigue observed in our study shows that routine assessment should be implemented and that further research is needed to clarify its underlying mechanisms and develop effective, targeted treatments for patients with VLD. Health literacy, which is often challenging in chronic diseases, positively predicts both mental and physical HRQoL.^53^ Furthermore, psychological interventions should be considered because of the psychological burden of VLD. Group support therapies appear to reduce depression and anxiety in patients with severe liver disease.^54^^,^^55^ Although therapeutic education programs have been implemented in Europe, they focus primarily on anticoagulation and medical management. Certain promising trials also suggest that physical activity improves fatigue.^56^ Thus, expanding programs to include mental health, physical activity, and social support could improve patient outcomes.

### Strengths and limitations

This study has several strengths. First, it is the largest multicentre study of PROs in patients with VLD, involving expert centres from three European countries. Despite the low prevalence of NC-PVT and BCS, the statistical power of our study is good because of the sample size, adding valuable insight into this underexplored population. Second, our approach integrates both clinical, sociodemographic, and socioeconomic data, providing a comprehensive perspective on factors influencing HRQoL. Third, comparisons with the general population were strengthened by indirect standardisation for age, gender, and educational level, increasing the validity of our external analyses by controlling for relevant confounders. Finally, given the large sample size, the acceptable response rate, the standardised outcome assessments, and the prospective collection of clinical data, the data from this observational study could serve as a benchmark of country-specific values for planning and interpreting future clinical trials in these rare diseases.

However, this study has certain limitations. First, the strength in the associations with some of the investigated factors could be biased because of the under-representation of younger individuals with low educational levels. The limited number of patients included in Spain and their high economic status might limit comparison with normative EQ-5D-5L and PHQ-8 values. Second, differences in clinical characteristics between respondents and nonrespondents suggest a nonrandom participation. Indeed, respondents had slightly more complications compared with nonrespondents, which might be because of more complex disease cases, making it more difficult to generalize our findings. Third, the cross-sectional design of the study also prevents any assessment of changes in HRQoL over time or inference of causal relationships. Fourth, although only 488 patients completed all three PROs, the overall response rate of ∼50% remains acceptable and similar to previously reported rates in epidemiological studies.^57^ Fifth, although we found a strong association between financial difficulties and the three PROs, it was not possible to determine whether financial difficulties were VLD related. Sixth, although the fatigue impact scale has been validated for the evaluation of fatigue in several liver diseases,^23^ the use of its modified short version (MFIS-5) validated for multiple sclerosis has not yet been validated in liver or rare liver diseases. Seventh, because we performed complete-case analyses, patients with missing data for the adjustment variables were excluded. This might have led to a selection bias if the missing data were not completely at random. Finally, despite the relatively large sample size of these rare conditions, the statistical power remains limited to detect associations among less prevalent clinical variables (e.g. history of overt HE, APS, PNH, or Behcet’s disease) even if the events-per-variable ratio were acceptable for each outcome.

## Conclusion

This multicentre European study shows the substantial burden of impaired HRQoL, fatigue, and depressive symptoms in patients with VLD, especially in women and economically vulnerable individuals. Although clinical factors, such as a history of overt HE and self-reported comorbidities, have a role, the main drivers of PRO impairment appear to be social. These findings emphasise the need for a comprehensive, patient-centred approach to the management of VLD, including the screening and management of encephalopathy, psychological assessment, and management of fatigue determinants as well as specific VLD therapeutic care, while focusing on the most vulnerable populations. Future interventions should integrate therapeutic education programs that address not only disease-specific knowledge and treatment adherence, but also mental health and HRQoL to improve long-term outcomes.

## Abbreviations

aCoeff, (adjusted) coefficient; aOR, adjusted odds ratio; APS, antiphospholipid syndrome; BCS, Budd-Chiari syndrome; HE, hepatic encephalopathy; HRQoL, health-related quality of life; ISCED, International Standard Classification of Education; MFIS-5, Modified Fatigue Impact Scale - short form; MPL, myeloproliferative leukaemia; NC-PVT, non-cirrhotic portal vein thrombosis; PH, portal hypertension; PHQ-8, Patient Health Questionnaire-8; PHQ-9, Patient Health Questionnaire-9; PNH, paroxysmal nocturnal haemoglobinuria; PROs, patient-reported outcomes; PVT, portal vein thrombosis; SIR, standardised incidence rate; TIPS, transjugular intrahepatic porto-systemic shunt; VALDIG, Vascular Liver Disease Group; VLD, vascular liver disease.

## Authors’ contributions

Conceptualization: AD, APl, VHG, AB, ADG. Data collection: VHG, LE, AB, AA, ADG, APa, PER, TK, AD, APl. Methodology, validation: AD, APl, CR. Data curation, formal analysis: CR. Project administration and funding acquisition: AD. Writing of the first draft of the manuscript: AD, APl, CR. Reviewed the paper: all authors. Approved the final version of the manuscript, including the authorship list: all authors.

## Data availability

Data supporting the findings of this study are available upon request from the scientific committee of the LIVES project (contact: agnes.dumas@inserm.fr). The data are not publicly available for privacy and ethical restrictions.

## Financial support

This study is part of the LIVES (Quality of life of patients living with vascular LIVEr diseaseS) project, which has received funding from the European Union’s Horizon 2020 Research and Innovation Programme under the EJP RD COFUND-EJP N° 825575, from the ANR (French National Research Agency), the SNF (Swiss National Science Foundation), the Instituto de Salud Carlos III, and the FWO (Fonds Wetenschappelijk Onderzoek – Vlaanderen). This article is also based on work from COST Action EURO-VALDI-NET, CA23146, supported by COST (European Cooperation in Science and Technology). This work was supported as part of the national plan for rare diseases by the French Ministry of Health.

## Conflicts of interest

The authors declare no potential conflicts of interest with respect to the research, authorship, and/or publication of this article.

Please refer to the accompanying ICMJE disclosure forms for further details.
