# Supplementary material for: Fatigue, depression, and impaired health-related quality of life in patients with vascular liver diseases: A multicentric European study
Source: JHEP Rep. 2026 Apr 17;8(8):101861. doi: 10.1016/j.jhepr.2026.101861 (PMC13380109; doi:10.1016/j.jhepr.2026.101861)
Supplement: Multimedia component 1 [file mmc1.pdf]

Fatigue, depression, and impaired health-related quality of life in patients with vascular liver diseases: A multicentric European study

Clémence Ramier, Virginia Hernandez-Gea, Laure Elkrief, Annalisa Berzigotti, Andrea De Gottardi, Antonina Antonenko, Audrey Payancé, Pierre-Emmanuel Rautou, Terhi Kangas, Hadewijch Vandenheede, Katrien Vanthomme, Gaël Brulé, Agnes Dumas, Aurélie Plessier, VALDIG consortium

Table of contents

Fig. S1. 2

Table S1. 3

Table S2 6

Table S3 8

Table S4 11

Table S5 12

Fig. S1. Precision about self-reported comorbidities.

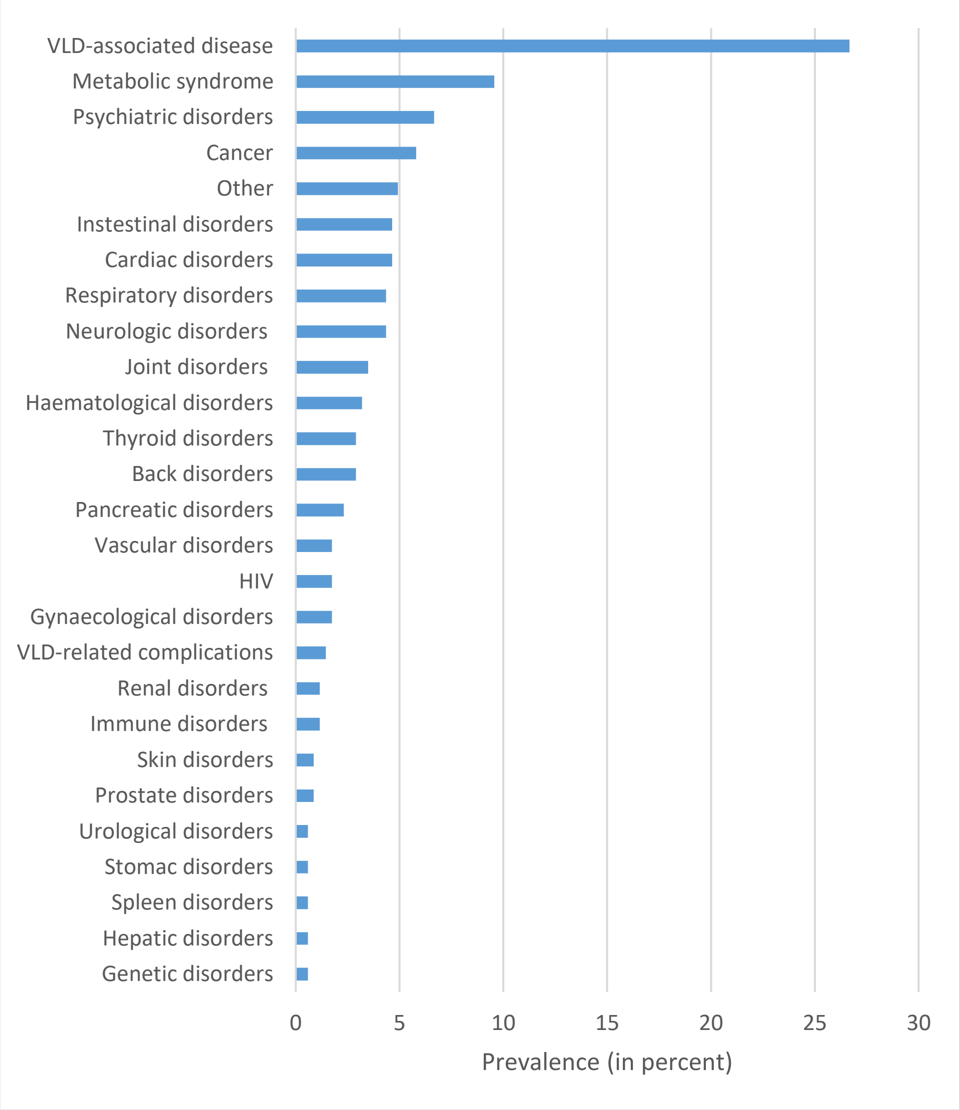

VLD, vascular liver disease.

Table S1. Questionnaires used for the three PROs: HRQoL, fatigue and depressive symptoms.

**EQ-5D-5L** [17] for health-related quality of life	*In the following questions, please choose ONE box, the one that best describes your health TODAY.*<br>**MOBILITY**<br>□ I have no problem getting around on foot<br>□ I have minor problems getting around on foot<br>□ I have moderate problems getting around on foot<br>□ I have severe problems getting around on foot<br>□ I am unable to get around on foot<br>**PERSONAL AUTONOMY**<br>□ I have no problem washing or dressing myself<br>□ I have minor problems washing or dressing myself<br>□ I have moderate problems washing or dressing myself.<br>□ I have severe problems washing or dressing myself<br>□ I am unable to wash or dress myself.<br>**CURRENT ACTIVITIES** (e.g. work, study, housework, family or leisure activities)<br>□ I have no problem carrying out my day-to-day activities<br>□ I have minor problems carrying out my day-to-day activities<br>□ I have moderate problems carrying out my day-to-day activities<br>□ I have severe problems carrying out my day-to-day activities<br>□ I am unable to carry out my day-to-day activities<br>**PAIN/DISCOMFORT**<br>□ I have no pain or discomfort<br>□ I have pain or slight discomfort<br>□ I have moderate pain or discomfort<br>□ I have severe pain or discomfort<br>□ I have extreme pain or discomfort<br>**ANXIETY/DEPRESSION**<br>□ I'm not anxious or depressed<br>□ I'm slightly anxious or depressed<br>□ I am moderately anxious or depressed<br>□ I am severely anxious or depressed<br>□ I'm extremely anxious or depressed
**Modified Fatigue Impact Scale-short form (MFIS-5)** [21] for fatigue	*Below is a list of statements that describe the effects of fatigue. Please read each statement carefully and tick the one that best indicates how often fatigue has affected you over the last 4 weeks.*<br>*Because of my fatigue over the last four weeks...*<br>**I was less alert**<br>□ Never<br>□ Rarely<br>□ Sometimes<br>□ Often<br>□ Almost always<br>**I was limited in my ability to do things away from home**<br>□ Never<br>□ Rarely<br>□ Sometimes<br>□ Often<br>□ Almost always<br>**I find it hard to sustain physical effort for long periods of time**<br>□ Never

	□ Rarely<br>□ Sometimes<br>□ Often<br>□ Almost always<br>**I was less able to carry out tasks requiring physical effort**<br>□ Never<br>□ Rarely<br>□ Sometimes<br>□ Often<br>□ Almost always<br>**I found it hard to concentrate**<br>□ Never<br>□ Rarely<br>□ Sometimes<br>□ Often<br>□ Almost always
**Patient Questionnaire-8 (PHQ-8)** [24] for depressive symptoms	*Over the last 2 weeks, how often have you been bothered by the following problems?*<br>**Little interest or pleasure in doing things**<br>□ Never<br>□ Several days<br>□ More than half the time<br>□ Almost every day<br>**Being sad, depressed or in despair**<br>□ Never<br>□ Several days<br>□ More than half the time<br>□ Almost every day<br>**Difficulty falling asleep or staying asleep, or sleeping too much**<br>□ Never<br>□ Several days<br>□ More than half the time<br>□ Almost every day<br>**Feeling tired or lacking energy**<br>□ Never<br>□ Several days<br>□ More than half the time<br>□ Almost every day<br>**Having little appetite or eating too much**<br>□ Never<br>□ Several days<br>□ More than half the time<br>□ Almost every day<br>**Having a low opinion of yourself, or feeling that you are rubbish, or that you have disappointed your family or yourself**<br>□ Never<br>□ Several days<br>□ More than half the time<br>□ Almost every day<br>**Having difficulty concentrating, for example, when reading the newspaper or watching television**<br>□ Never

	□ Several days<br>□ More than half the time<br>□ Almost every day<br>**Moving or speaking so slowly that others might have noticed. Or, on the contrary, being so agitated that you found it harder than usual to hold still.**<br>□ Never<br>□ Several days<br>□ More than half the time<br>□ Almost every day

Table S2. Characteristics of eligible patients and comparison between respondents and non-respondents to all the three PROs.

**Characteristics** (%. of missing values)	**Non-respondents** (N=648) N (%) or median [IQR]	**Respondents** (N=488) N (%) or median [IQR]	P-value[1]
**SOCIODEMOGRAPHIC AND ECONOMIC CHARACTERISTICS**			
**Gender** (0.1)			
Men	372 (57.4)	259 (53.1)	0.146
Women	276 (42.6)	229 (46.9)	
**Age** (0.4)	52 [40 – 64]	53 [42.5 – 63.5]	0.297
**DIAGNOSIS-SPECIFIC CHARACTERISTICS**			
**Diagnosis** (0.3)			
PVT	519 (80.3)	366 (75.0)	0.031
BCS	127 (19.7)	122 (25.0)	
**Age at diagnosis** (0)	41 [30 – 54]	43 [31 – 54]	0.399
**Time since diagnosis** (0)	7.7 [4.4 – 13.2]	8.7 [4.6 – 14.3]	0.058
**Time since diagnosis** (0)			
≥2 years	606 (93.4)	453 (92.8)	0.718
<2 years	43 (6.6)	35 (7.2)	
**HISTORY OF VLD-RELATED COMPLICATIONS**			
**Abdominal pain** (10.1)	385 (68.9)	331 (71.5)	0.363
**Ascites** (7.2)	424 (71.6)	345 (74.5)	0.294
**Oesophageal varices** (17.9)	296 (61.7)	242 (53.4)	0.011
**Gastric varices** (22.0)	47 (10.8)	55 (12.2)	0.525
**Gastrointestinal bleeding** (8.0)	87 (14.9)	76 (16.5)	0.492
**Hepatic encephalopathy** (16.5)	28 (5.4)	23 (5.4)	0.995
**Thrombotic event** (1.1)	213 (33.2)	206 (42.6)	0.001
**Liver cancer** (18.4)	14 (2.8)	7 (1.6)	0.234
**HISTORY OF ASSOCIATED DISEASES**			
**Diabetes** (8.7)	56 (9.7)	34 (7.4)	0.179
**Arterial hypertension** (8.8)	90 (15.6)	75 (16.3)	0.757
**Anaemia** (10.6)	54 (9.5)	47 (10.4)	0.632
**Myeloproliferative leukaemia** (9.4)	128 (22.3)	127 (27.8)	0.044
**Antiphospholipid syndrome** (13.5)	28 (5.2)	19 (4.3)	0.512
**Paroxysmal nocturnal haemoglobinuria** (20.5)	5 (1.0)	12 (3.0)	0.030
**Behcet's disease** (14.1)	15 (2.8)	18 (4.0)	0.308
**Factor V Leiden mutation** (14.1)	35 (6.5)	39 (8.9)	0.162
**Prothrombin G20210A mutation** (15.8)	28 (5.4)	38 (8.7)	0.042
**LAST AVAILABLE PROGNOSIS SCORES FOR BCS**			
**Child-Pugh score** (20.3)			0.036
PVT	519 (84.3)	366 (77.4)	
Class A	26 (4.2)	29 (6.1)	
Class B	61 (9.9)	69 (14.6)	

**Characteristics** (%. of missing values)	**Non-respondents** (N=648) N (%) or median [IQR]	**Respondents** (N=488) N (%) or median [IQR]	P-value[1]
Class C	10 (1.6)	9 (1.9)	
**Rotterdam score** (21.0)			0.022
PVT	519 (84.3)	366 (77.4)	
Class I	43 (7.0)	34 (7.3)	
Class II	24 (3.9)	31 (6.6)	
Class III	28 (4.6)	36 (7.7)	
**HISTORY OF THERAPEUTIC STRATEGIES**			
**Interventional radiology or surgical procedures[2]** (18.2)	85 (16.9)	79 (18.5)	0.543
**Liver transplant** (18.4)	14 (2.8)	6 (1.4)	0.145
**Anticoagulation therapy** (0)	475 (73.2)	390 (79.9)	0.008
**Diuretic therapy** (0)	62 (9.6)	55 (11.3)	0.346
**Antiplatelet therapy** (0)	33 (5.1)	25 (5.1)	0.977
**Beta-blocker therapy** (0)	131 (20.2)	103 (21.1)	0.704

BCS, Budd-Chiari syndrome; IQR, interquartile range; PVT, portal vein thrombosis

[1]Chi-squared or exact Fisher tests for categorical variables and Kruskal-Wallis test for continuous variables. Level of significance at 5%.

[2]Interventional radiology or surgical procedures included angioplasty, stenting, TIPS or shunt surgery.

Table S3. Factors associated with HRQoL, fatigue and depressive symptoms in univariable analyses (Linear and logistic regressions, N=488)

Explanatory variables	HRQoL		Fatigue		Depressive symptoms	
	Coeff [95% CI]	P-value[1]	Coeff [95% CI]	P-value[1]	OR [95% CI]	P-value[2]
**SOCIODEMOGRAPHIC AND ECONOMIC CHARACTERISTICS**						
**Gender**						
Men	Ref.		Ref.		Ref.	
Women	-0.04 [-0.07;0.02]	0.001	1.44 [0.51;2.37]	0.003	2.36 [1.55;3.61]	<0.001
**Age**	-0.00 [-0.00;0.00]	0.919	-0.01 [-0.04;0.02]	0.636	0.98 [0.97;1.00]	0.009
**Country of birth**						
EU	Ref.		Ref.		Ref.	
Non-EU	-0.05 [-0.10;-0.00]	0.041	2.12 [0.67;3.56]	0.004	2.38 [1.37;4.12]	0.002
**Living with a partner**	0.02 [-0.01;0.05]	0.242	-0.22 [-1.23;0.79]	0.668	0.86 [0.55;1.33]	0.490
**Educational level**		**0.593**		**0.219**		**0.356**
Low	-0.04 [-0.11;0.04]	0.367	2.05 [-0.27;4.37]	0.083	0.76 [0.28;2.09]	0.593
Moderate	-0.01 [-0.04;0.02]	0.539	0.24 [-0.72;1.20]	0.622	1.30 [0.85;1.99]	0.226
High	Ref.		Ref.		Ref.	
**Financial difficulties**		<0.001		<0.001		<0.001
No	Ref.		Ref.		Ref.	
Few	-0.03 [-0.05;-0.00]	0.037	1.73 [0.73;2.72]	0.001	2.02 [1.22;3.36]	0.006
A lot	-0.12 [-0.17;-0.06]	<0.001	4.52 [3.13;5.92]	<0.001	5.67 [3.01;10.71]	<0.001
**DIAGNOSIS-SPECIFIC CHARACTERISTICS**						
**Diagnosis**						
PVT	Ref.		Ref.		Ref.	
BCS	0.01 [-0.02;0.04]	0.582	0.16 [-0.93;1.25]	0.772	0.99 [0.61;1.59]	0.952
**Age at diagnosis**	0.00 [-0.00;0.00]	0.444	-0.02 [-0.05;0.01]	0.144	0.99 [0.97;1.00]	0.020
**Time since diagnosis**	-0.00 [-0.00;0.00]	0.506	0.03 [-0.03;0.10]	0.288	1.00 [0.98;1.03]	0.723
**Time since diagnosis**						
≥2 years	Ref.		Ref.		Ref.	
<2 years	0.05 [0.00;0.10]	0.043	-0.96 [-2.78;0.85]	0.298	0.61 [0.25;1.50]	0.281
**HISTORY OF VLD-RELATED COMPLICATIONS**						
**Impaired fertility**	-0.05 [-0.10;0.01]	0.074	1.42 [0.03;2.81]	0.046	1.92 [1.09;3.37]	0.024

Explanatory variables	HRQoL		Fatigue		Depressive symptoms	
	Coeff [95% CI]	P-value[1]	Coeff [95% CI]	P-value[1]	OR [95% CI]	P-value[2]
**Abdominal pain**	0.00 [-0.03;0.03]	0.790	0.40 [-0.67;1.47]	0.463	0.85 [0.54;1.34]	0.487
**Ascites**	0.00 [-0.03;0.03]	0.964	0.83 [-0.25;1.91]	0.134	0.97 [0.60;1.58]	0.915
**Oesophageal varices**	-0.00 [-0.03;0.03]	0.840	0.64 [-0.33;1.60]	0.196	0.87 [0.57;1.33]	0.530
**Gastric varices**	0.00 [-0.04;0.04]	0.984	1.02 [-0.41;2.46]	0.162	0.81 [0.41;1.59]	0.536
**Gastrointestinal bleeding**	-0.03 [-0.07;0.01]	0.136	1.30 [-0.02;2.62]	0.054	1.41 [0.82;2.43]	0.218
**Hepatic encephalopathy**	-0.07 [-0.12;-0.01]	0.030	2.97 [0.63;5.30]	0.013	1.67 [0.69;4.06]	0.258
**Thrombotic event**	-0.01 [-0.04;0.01]	0.366	-0.57 [-1.52;0.38]	0.238	0.98 [0.64;1.48]	0.916
**HISTORY OF ASSOCIATED DISEASES**						
**Self-reported comorbidities**	-0.06 [-0.09;-0.03]	<0.001	2.65 [1.70;3.60]	<0.001	1.60 [1.02;2.52]	0.042
**Diabetes**	-0.01 [-0.06;0.04]	0.754	-0.10 [-2.07;1.87]	0.918	0.63 [0.26;1.58]	0.328
**Arterial hypertension**	0.01 [-0.03;0.05]	0.596	-0.83 [-2.06;0.40]	0.186	0.65 [0.35;1.22]	0.183
**Anaemia**	-0.06 [-0.12;-0.00]	0.048	2.07 [0.35;3.78]	0.018	1.86 [0.98;3.53]	0.056
**Myeloproliferative leukaemia**	0.00 [-0.03;0.03]	0.835	0.90 [-0.13;1.93]	0.088	1.00 [0.63;1.61]	0.992
**Antiphospholipid syndrome**	-0.03 [-0.14;0.07]	0.503	-1.05 [-3.98;1.88]	0.483	2.77 [1.09;7.01]	0.032
**Paroxysmal nocturnal haemoglobinuria**	-0.02 [-0.16;0.13]	0.816	1.51 [-2.00;5.01]	0.398	0.58 [0.13;2.72]	0.493
**Behcet's disease**	-0.02 [-0.08;0.04]	0.595	-0.32 [-2.58;1.94]	0.778	0.84 [0.27;2.61]	0.765
**Factor V Leiden mutation**	0.00 [-0.04;0.05]	0.852	-0.64 [-2.32;1.04]	0.458	0.15 [0.03;0.62]	0.009
**Prothrombin G20210A mutation**	0.02 [-0.03;0.06]	0.461	-0.56 [-2.03;0.91]	0.457	0.65 [0.28;1.52]	0.316
**LAST AVAILABLE PROGNOSIS SCORES FOR BCS**						
**Child-Pugh score**		**0.521**		**0.205**		**0.505**
PVT	Ref.		Ref.		Ref.	
Class A	0.03 [-0.02;0.06]	0.254	1.12 [-1.02 ;3.26]	0.305	1.59 [0.71;3.55]	0.257
Class B	0.01 [-0.04;0.05]	0.772	-0.29 [-1.65;1.07]	0.672	0.91 [0.50;1.68]	0.767
Class C	-0.04 [-0.12;0.05]	0.382	2.20 [-0.19;4.61]	0.071	0.38 [0.05;3.07]	0.362
**Rotterdam score**		**0.548**		**0.922**		**0.997**
PVT	Ref.		Ref.		Ref.	
Class I	0.01 [-0.05 ;0.06]	0.827	0.52 [-1.35;2.39]	0.586	0.93 [0.41;2.13]	0.863
Class II	0.03 [-0.01;0.06]	0.178	0.30 [-1.77;2.38]	0.775	1.05 [0.45;2.43]	0.907

Explanatory variables	HRQoL		Fatigue		Depressive symptoms	
	Coeff [95% CI]	P-value[1]	Coeff [95% CI]	P-value[1]	OR [95% CI]	P-value[2]
Class III	-0.01 [-0.07;0.05]	0.695	0.38 [-1.39;2.14]	0.675	1.01 [0.46;2.22]	0.986
**Clichy score**		**0.916**		**0.673**		**0.529**
PVT	Ref.		Ref.		Ref.	
Class I	0.01 [-0.03 ;0.04]	0.725	0.55 [-0.74;1.84]	0.400	1.18 [0.69;2.02]	0.550
Class II	0.01 [-0.04;0.05]	0.786	-0.19 [-2.20;1.81]	0.850	0.60 [0.20;1.82]	0.370
**HISTORY OF THERAPEUTIC STRATEGIES**						
**Interventional radiology or surgical procedures[3]**	-0.04 [-0.09;0.00]	0.063	2.04 [0.75;3.31]	0.002	1.42 [0.83;2.44]	0.203
**Anticoagulation therapy**	0.04 [0.01;0.07]	0.005	-1.93 [-3.06;-0.80]	0.001	0.57 [0.35;0.93]	0.024
**Diuretic therapy**	0.01 [-0.03;0.05]	0.495	1.10 [-0.35;2.54]	0.137	0.64 [0.31;1.32]	0.231
**Antiplatelet therapy**	0.03 [-0.01;0.06]	0.166	0.08 [2.67;1.82]	0.933	1.76 [0.76;4.10]	0.188
**Beta-blocker therapy**	-0.02 [-0.05;0.02]	0.310	1.01 [-0.11;2.14]	0.078	0.90 [0.54;1.51]	0.693
**Follow-up in an anticoagulant clinic**	-0.01 [-0.04;0.02]	0.603	0.60 [-0.52;1.71]	0.291	0.97 [0.60;1.56]	0.904

BCS, Budd-Chiari syndrome; CI, confidence interval; Coeff, adjusted coefficient; EU, European Union; IQR, interquartile range; OR, adjusted odds ratio; PVT, portal vein thrombosis

[1]Univariable linear regression with a significant level set at 5%.

[2]Univariable logistic regression with a significant level set at 5%.

[3]Interventional radiology or surgical procedures included angioplasty, stenting, TIPS or shunt surgery.

Table S4. Sensitivity analyses on factors associated with each domain of the HRQoL (multivariable linear regression, N=448)

Explanatory variables	Mobility[1] (N=444)		Self-care[1] (N=463)		Usual activities[1] (N=444)		Pain/Discomfort[1] (N=444)		Depression/Anxiety[1] (N=389)	
	aCoeff [95% CI]	P-value	aCoeff [95% CI]	P-value	aCoeff [95% CI]	P-value	aCoeff [95% CI]	P-value	aCoeff [95% CI]	P-value
**Gender**										
Men	Ref.				Ref.		Ref.		Ref.	
Women	0.02 [0.00;0.04]	0.026			0.02 [0.00;0.03]	0.046	0.02 [0.00;0.03]	0.048	0.03 [0.01;0.05]	0.005
**Age**	0.00 [0.00;0.00]	0.002								
**Country of birth**										
EU	Ref.				Ref.		Ref.			
Non-EU	0.04 [0.01;0.06]	0.004			0.03 [0.01;0.06]	0.015	0.04 [0.01;0.06]	0.006		
**Financial difficulties**		**<0.001**				**<0.001**		**<0.001**		**0.035**
No	Ref.				Ref.		Ref.		Ref.	
Few	0.01 [-0.01;0.02]	0.480			0.02 [0.01;0.04]	0.004	0.02 [-0.00;0.04]	0.056	0.02 [0.00;0.04]	0.033
A lot	0.06 [0.03;0.09]	<0.001			0.08 [0.05;0.11]	<0.001	0.06 [0.03;0.09]	<0.001	0.04 [0.00;0.07]	0.036
**Time since diagnosis**										
≥2 years							Ref.			
<2 years							-0.04 [-0.07;-0.02]	0.002		
**Ascites**			0.01 [0.00;0.02]	<0.001						
**Hepatic encephalopathy**									0.06 [0.01;0.12]	0.031
**Self-reported comorbidities**	0.03 [0.01;0.04]	<0.001			0.03 [0.02;0.05]	<0.001	0.04 [0.02;0.06]	<0.001	0.03 [0.01;0.05]	0.001
**Arterial hypertension**									-0.03 [-0.05;-0.00]	0.034
**Anticoagulation therapy**									-0.03 [-0.06;-0.00]	0.044
**Antiplatelet therapy**	-0.03 [-0.06;-0.01]	0.008	-0.01 [-0.01;-0.01]	<0.001			-0.03 [-0.06;-0.00]	0.033		

BCS, Budd-Chiari syndrome; (a)Coeff, (adjusted) coefficient; CI, confidence interval; EU, European Union; PVT, portal vein thrombosis

Significant level set at 5%.

[1]Each domain was categorized from 1 (no problem) to 5 (extreme problem).

Table S5. Sensitivity analyses on factors associated with each domain of the fatigue (multivariable linear regression, N=448)

Explanatory variables	Physical[1] (N=420)		Cognitive[1] (N=388)		Psychosocial[2] (N=367)	
	aCoeff [95% CI]	P-value	aCoeff [95% CI]	P-value	aCoeff [95% CI]	P-value
**Gender**						
Men	Ref.		Ref.		Ref.	
Women	0.53 [0.09;0.98]	0.018	0.45 [0.06;0.85]	0.023	0.29 [0.06;0.53]	0.015
**Country of birth**						
EU	Ref.				Ref.	
Non-EU	0.87 [0.21;1.52]	0.010			0.68 [0.31;1.06]	<0.001
**Financial difficulties**		**<0.001**		**0.003**		**0.002**
No	Ref.		Ref.		Ref.	
Few	0.60 [0.12;1.07]	0.014	0.29 [-0.14;0.73]	0.189	0.20 [-0.04;0.45]	0.160
A lot	1.73 [1.03;2.43]	<0.001	1.05 [0.44;1.65]	0.001	0.80 [0.37;1.24]	<0.001
**Abdominal pain**			0.44 [0.04;0.84]	0.032		
**Ascites**	0.51 [0.05;0.97]	0.030				
**Hepatic encephalopathy**			0.92 [0.13;1.70]	0.023		
**Self-reported comorbidities**	1.19 [0.74;1.63]	<0.001	0.88 [0.51;1.26]	<0.001	0.49 [0.25;0.72]	<0.001
**Arterial hypertension**			-0.63 [-1.12;-0.15]	0.010		
**Paroxysmal nocturnal haemoglobinuria**					1.04 [0.29;1.80]	0.007
**Interventional radiology or surgical procedures[3]**			0.56 [0.06;1.06]	0.027		
**Anticoagulation therapy**	-0.63 [-1.16;-0.10]	0.019	-0.82 [-1.37;-0.27]	0.004	-0.54 [-0.84;-0.23]	0.001

BCS, Budd-Chiari syndrome; (a)Coeff, (adjusted) coefficient; CI, confidence interval; EU, European Union; PVT, portal vein thrombosis

Significant level set at 5%.

[1]Physical and cognitive domains ranged from 0 (disorders never felt) to 8 (disorders almost always felt).

[2]Psychosocial domain ranged from 0 (disorder never felt) to 4 (disorder almost always felt).

[3]Interventional radiology or surgical procedures included angioplasty, stenting, TIPS or shunt surgery.
